# Sequencing and analysis of an Irish human genome

**DOI:** 10.1186/gb-2010-11-9-r91

**Published:** 2010-09-07

**Authors:** Pin Tong, James GD Prendergast, Amanda J Lohan, Susan M Farrington, Simon Cronin, Nial Friel, Dan G Bradley, Orla Hardiman, Alex Evans, James F Wilson, Brendan Loftus

**Affiliations:** 1 Conway Institute, University College Dublin, Belfield, Dublin 4, Ireland; 2 MRC Human Genetics Unit, Western General Hospital, Crewe Road, Edinburgh, EH4 2XU, UK; 3 Colon Cancer Genetics Group and Academic Coloproctology, Institute of Genetics and Molecular Medicine, University of Edinburgh, Edinburgh, EH4 2XU, UK; 4 Department of Clinical Neurological Sciences, Royal College of Surgeons in Ireland, Dublin 2, Ireland; 5 School of Mathematical Sciences, University College Dublin, Belfield, Dublin 4, Ireland; 6 Smurfit Institute of Genetics, Trinity College Dublin, Dublin 2, Ireland; 7 Department of Neurology, Beaumont Hospital and Trinity College Dublin, Beaumont Road, Dublin 9, Ireland; 8 School of Agriculture, Food Science and Veterinary Medicine, University College Dublin, Belfield, Dublin 4, Ireland; 9 Centre for Population Health Sciences, University of Edinburgh, Teviot Place, Edinburgh, EH8 9AG, UK

## Abstract

**Background:**

Recent studies generating complete human sequences from Asian, African and European subgroups have revealed population-specific variation and disease susceptibility loci. Here, choosing a DNA sample from a population of interest due to its relative geographical isolation and genetic impact on further populations, we extend the above studies through the generation of 11-fold coverage of the first Irish human genome sequence.

**Results:**

Using sequence data from a branch of the European ancestral tree as yet unsequenced, we identify variants that may be specific to this population. Through comparisons with HapMap and previous genetic association studies, we identified novel disease-associated variants, including a novel nonsense variant putatively associated with inflammatory bowel disease. We describe a novel method for improving SNP calling accuracy at low genome coverage using haplotype information. This analysis has implications for future re-sequencing studies and validates the imputation of Irish haplotypes using data from the current Human Genome Diversity Cell Line Panel (HGDP-CEPH). Finally, we identify gene duplication events as constituting significant targets of recent positive selection in the human lineage.

**Conclusions:**

Our findings show that there remains utility in generating whole genome sequences to illustrate both general principles and reveal specific instances of human biology. With increasing access to low cost sequencing we would predict that even armed with the resources of a small research group a number of similar initiatives geared towards answering specific biological questions will emerge.

## Background

Publication of the first human genome sequence heralded a landmark in human biology [1]. By mapping out the entire genetic blueprint of a human, and as the culmination of a decade long effort by a variety of centers and laboratories from around the world, it represented a significant technical as well as scientific achievement. However, prior the publication, much researcher interest had shifted towards a 'post-genome' era in which the focus would move from the sequencing of genomes to interpreting the primary findings. The genome sequence has indeed prompted a variety of large scale post-genome efforts, including the encyclopedia of DNA elements (ENCODE) project [2], which has pointed towards increased complexity at the levels of the genome and transcriptome. Analysis of this complexity is increasingly being facilitated by a proliferation of sequence-based methods that will allow high resolution measurements of both and the activities of proteins that either transiently or permanently associate with them [3,4].

However, the advent of second and third generation sequencing technologies means that the landmark of sequencing an entire human genome for $1,000 is within reach, and indeed may soon be surpassed [5]. The two versions of the human genome published in 2001, while both seminal achievements, were mosaic renderings of a number of individual genomes. Nevertheless, it has been clear for some time that sequencing additional representative genomes would be needed for a more complete understanding of genomic variation and its relationship to human biology. The structure and sequence of the genome across human populations is highly variable, and generation of entire genome sequences from a number of individuals from a variety of geographical backgrounds will be required for a comprehensive assessment of genetic variation. SNPs as well as insertions/deletions (indels) and copy number variants all contribute to the extensive phenotypic diversity among humans and have been shown to associate with disease susceptibility [6]. Consequently, several recent studies have undertaken to generate whole genome sequences from a variety of normal and patient populations [7]. Similarly, whole genome sequences have recently been generated from diverse human populations, and studies of genetic diversity at the population level have unveiled some interesting findings [8]. These data look to be dramatically extended with releases of data from the 1000 Genomes project [9]. The 1000 Genomes project aims to achieve a nearly complete catalog of common human genetic variants (minor allele frequencies > 1%) by generating high-quality sequence data for > 85% of the genome for 10 sets of 100 individuals, chosen to represent broad geographic regions from across the globe. Representation of Europe will come from European American samples from Utah and Italian, Spanish, British and Finnish samples.

In a recent paper entitled 'Genes mirror geography within Europe' [10], the authors suggest that a geographical map of Europe naturally arises as a two-dimensional summary of genetic variation within Europe and state that when mapping disease phenotypes spurious associations can arise if genetic structure is not properly accounted for. In this regard Ireland represents an interesting case due to its position, both geographically and genetically, at the western periphery of Europe. Its population has also made disproportionate ancestral contributions to other regions, particularly North America and Australia. Ireland also displays a maximal or near maximal frequency of alleles that cause or pre-dispose to a number of important diseases, including cystic fibrosis, hemochromatosis and phenylketonuria [11]. This unique genetic heritage has long been of interest to biomedical researchers and this, in conjunction with the absence of an Irish representative in the 1000 Genomes project, prompted the current study to generate a whole genome sequence from an Irish individual. The resulting sequence should contain rare structural and sequence variants potentially specific to the Irish population or underlying the missing heritability of chronic diseases not accounted for by the common susceptibility markers discovered to date [12]. In conjunction with the small but increasing number of other complete human genome sequences, we hoped to address a number of other broader questions, such as identifying key targets of recent positive selection in the human lineage.

## Results and discussion

### Data generated

The genomic DNA used in this study was obtained from a healthy, anonymous male of self-reported Irish Caucasian ethnicity of at least three generations, who has been genotyped and included in previous association and population structure studies [13-15]. These studies have shown this individual to be a suitable genetic representative of the Irish population (Additional file 1).

Four single-end and five paired-end DNA libraries were generated and sequenced using a GAII Illumina Genome Analyzer. The read lengths of the single-end libraries were 36, 42, 45 and 100 bp and those of the paired end were 36, 40, 76, and 80 bp, with the span sizes of the paired-end libraries ranging from 300 to 550 bp (± 35 bp). In total, 32.9 gigabases of sequence were generated (Table 1). Ninety-one percent of the reads mapped to a unique position in the reference genome (build 36.1) and in total 99.3% of the bases in the reference genome were covered by at least one read, resulting in an average 10.6-fold coverage of the genome.

**Table 1:** Read information

Data type	Library number	Number of reads	Number of mapped reads	Total bases (Gb)	Mapped base (Gb)	Effective depth
Single-end read	4	155,704,190	142,333,466	9.7	9.1	3.2
Paired-end read	5	324,936,690	297,787,256	23.2	21.2	7.4
Total	9	480,640,880	440,120,722	32.9	30.3	10.6

### SNP discovery and novel disease-associated variants

#### SNP discovery

**Figure 1:**
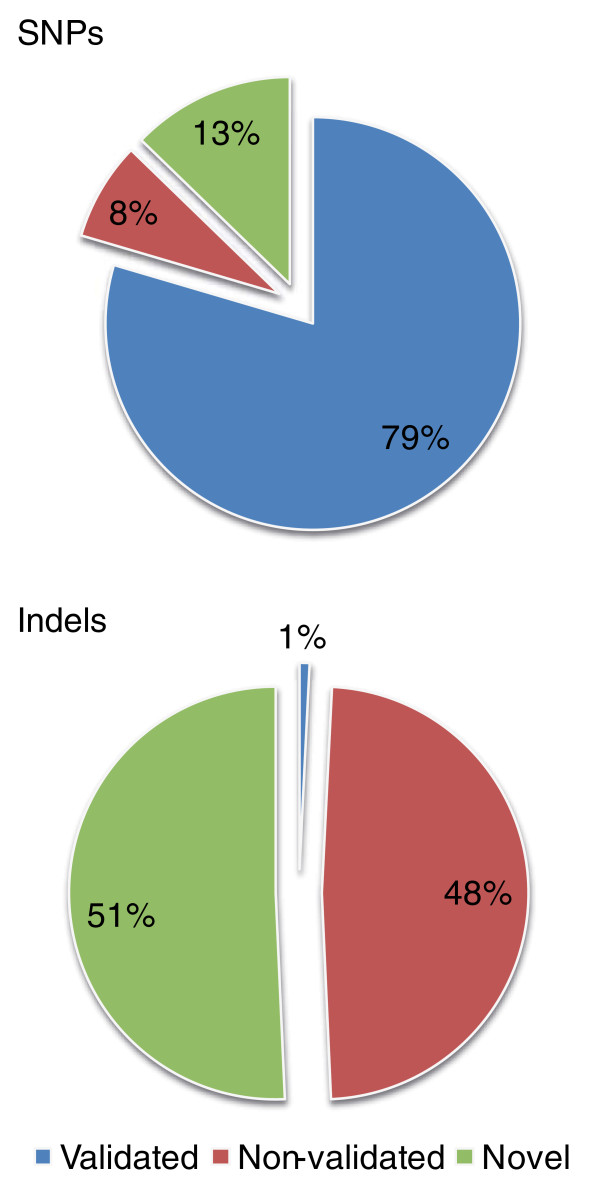
**Comparison of detected SNPs and indels to dbSNP130**. The dbSNP alleles were separated into validated and non-validated, and the detected variations that were not present in dbSNP were classified as novel.

Comparison with the reference genome identified 3,125,825 SNPs in the Irish individual, of which 87% were found to match variants in dbSNP130 (2,486,906 as validated and 240,791 as non-validated; Figure 1). The proportion of observed homozygotes and heterozygotes was 42.1% and 57.9%, respectively, matching that observed in previous studies [16]. Of those SNPs identified in coding regions of genes, 9,781 were synonymous, 10,201 were non-synonymous and 107 were nonsense. Of the remainder, 24,238 were located in untranslated regions, 1,083,616 were intronic and the remaining 1,979,180 were intergenic (Table 2). In order to validate our SNP calling approach (see Materials and methods) we compared genotype calls from the sequencing data to those obtained using a 550 k Illumina bead array. Of those SNPs successfully genotyped on the array, 98% were in agreement with those derived from the sequencing data with a false positive rate estimated at 0.9%, validating the quality and reproducibility of the SNPs called.

**Table 2:** Types of SNPs found

Consequence	Number of SNPs	% of SNPs
Essential_splice_site	135	0.0043
Stop_gained	107	0.0034
Stop_lost	23	0.0007
Non_synonymous_coding	10,201	0.3263
Splice_site	2,002	0.0640
Synonymous_coding	9,781	0.3129
Within_mature_mirna	30	0.0010
Within_non_coding_gene	16,512	0.5282
5prime_utr	4,599	0.1471
3prime_utr	19,639	0.6283
Intronic	1,083,616	34.6666
Other	1,979,180	63.3170

#### Disease-associated variants

Various disease-associated SNPs were detected in the sequence, but they are likely to be of restricted widespread value in themselves. However, a large proportion of SNPs in the Human Gene Mutation Database (HGMD) [17], genome-wide association studies (GWAS) [18] and the Online Mendelian Inheritance in Man (OMIM) database [19] are risk markers, not directly causative of the associated disease but rather in linkage disequilibrium (LD) with generally unknown SNPs that are. Therefore, in order to interrogate our newly identified SNPs for potential causative risk factors, we looked for those that appeared to be in LD with already known disease-associated (rather than disease-causing) variants. We identified 23,176 novel SNPs in close proximity (< 250 kb) to a known HGMD or genome-wide association study disease-associated SNP and where both were flanked by at least one pair of HapMap [20] CEU markers known to be in high LD. As the annotation of the precise risk allele and strand of SNPs in these databases is often incomplete, we focused on those positions, heterozygous in our individual, that are associated with a disease or syndrome. Of the 7,682 of these novel SNPs that were in putative LD of a HGMD or genome-wide association study disease-associated SNP heterozygous in our individual, 31 were non-synonymous, 14 were at splice sites (1 annotated as essential) and 1 led to the creation of a stop codon (Table S1 in Additional file 2).

This nonsense SNP is located in the macrophage-stimulating immune gene *MST1*, 280 bp 5' of a non-synonymous coding variant marker (rs3197999) that has been shown in several cohorts to be strongly associated with inflammatory bowel disease and primary sclerosing cholangitis [21-23]. Our individual was heterozygous at both positions (confirmed via resequencing; Additional files 3 and 4) and over 30 pairs of HapMap markers in high LD flank the two SNPs. The role of *MST1 *in the immune system makes it a strong candidate for being the gene in this region conferring inflammatory bowel disease risk, and it had previously been proposed that rs3197999 could itself be causative due to its potential impact on the interaction between the MST1 protein product and its receptor [22].

**Figure 2:**
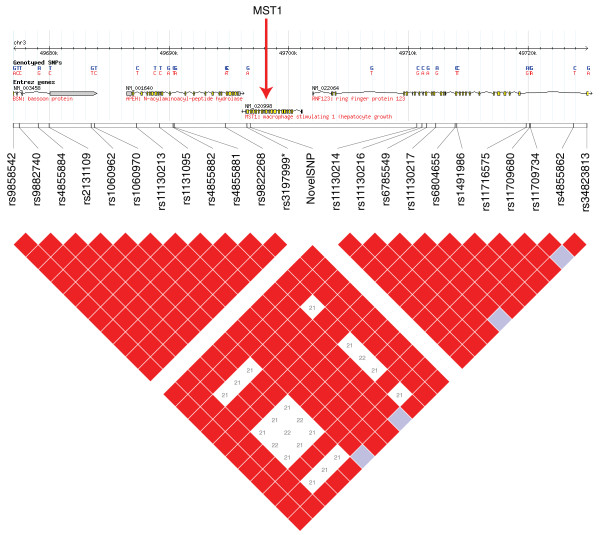
**The linkage disequilibrium structure in the immediate region of the *MST1 *gene**. Red boxes indicate SNPs in high LD. rs3197999, which has previously been associated with inflammatory bowel disease, and our novel nonsense SNP are highlighted in blue.

Importantly, the newly identified SNP 5' of rs3197999's position in the gene implies that the entire region 3' of this novel SNP would be lost from the protein, including the amino acid affected by rs3197999 (Figure 2). Therefore, although further investigation is required, there remains a possibility that this previously unidentified nonsense SNP is either conferring disease risk to inflammatory bowel disease marked by rs3197999, or if rs3197999 itself confers disease as previously hypothesized [22], this novel SNP is conferring novel risk via the truncation of the key region of the MST1 protein.

Using the SIFT program [24], we investigated whether those novel non-synonymous SNPs in putative LD with risk markers were enriched with SNPs predicted to be deleterious (that is, that affect fitness), and we indeed found an enrichment of deleterious SNPs as one would expect if an elevated number were conferring risk to the relevant disease. Of all 7,993 non-synonymous allele changes identified in our individual for which SIFT predictions could be successfully made, 26% were predicted to be deleterious. However, of those novel variants in putative LD with a disease SNP heterozygous in our individual, 56% (14 out of 25) were predicted to be harmful by SIFT (chi-square *P *= 6.8 × 10^-4^, novel non-synonymous SNPs in putative LD with risk allele versus all non-synonymous SNPs identified). This suggests that this subset of previously unidentified non-synonymous SNPs in putative LD with disease markers is indeed substantially enriched for alleles with deleterious consequences.

### Indels

Indels are useful in mapping population structure, and measurement of their frequency will help determine which indels will ultimately represent markers of predominately Irish ancestry. We identified 195,798 short indels ranging in size from 29-bp deletions to 20-bp insertions (see Materials and methods). Of these, 49.3% were already present in dbSNP130. Indels in coding regions will often have more dramatic impacts on protein translation than SNPs, and accordingly be selected against, and unsurprisingly only a small proportion of the total number of short indels identified were found to map to coding sequence regions. Of the 190 novel coding sequence indels identified (Table S2 Additional file 2), only 2 were at positions in putative LD with a heterozygous disease-associated SNP, of which neither led to a frameshift (one caused an amino acid deletion and one an amino acid insertion; Table S1 in Additional file 2).

### Population genetics

The DNA sample from which the genome sequence was derived has previously been used in an analysis of the genetic structure of 2,099 individuals from various Northern European countries and was shown to be representative of the Irish samples. The sample was also demonstrated to be genetically distinct from the core group of individuals genotyped from neighboring Britain, and the data are likely, therefore, to complement the upcoming 1000 Genomes data derived from British heritage samples (including CEU; Additional file 1).

**Figure 3:**
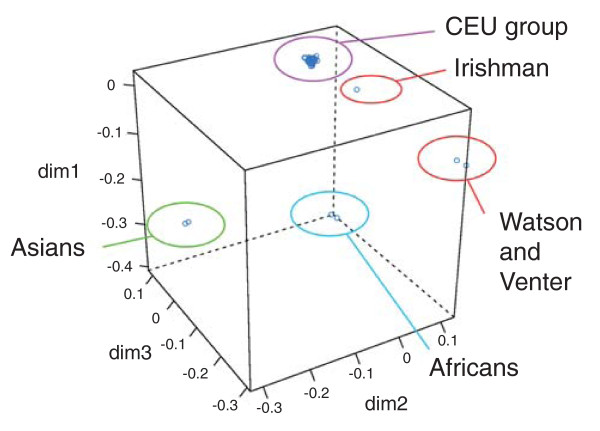
**Multidimensional scaling plot illustrating the Irish individual's relationship to the CEU HapMap individuals and other previously sequenced genomes**.

Non-parametric population structure analysis [25] was carried out to determine the positioning of our Irish individual relative to other sequenced genomes and the CEU HapMap dataset. As can be seen in Figure 3, as expected, the African and Asian individuals form clear subpopulations in this analysis. The European samples form three further subpopulations in this analysis, with the Irish individual falling between Watson and Venter and the CEU subgroup (of which individual NA07022 has been sequenced [26]). Therefore, the Irish genome inhabits a hitherto unsampled region in European whole-genome variation, providing a valuable resource for future phylogenetic and population genetic studies.

Y chromosome haplotype analysis highlighted that our individual belonged to the common Irish and British S145+ subgroup (JFW, unpublished data) of the most common European group R1b [27]. Indeed, S145 reaches its maximum global frequency in Ireland, where it accounts for > 60% of all chromosomes (JFW, unpublished data). None of the five markers defining known subgroups of R1b-S145 could be found in our individual, indicating he potentially belongs to an as yet undefined branch of the S145 group. A subset of the (> 2,141) newly discovered Y chromosome markers found in this individual is therefore likely to be useful in further defining European and Irish Y chromosome lineages.

Mapping of reads to the mitochondrial DNA (mtDNA) associated with UCSC reference build 36 revealed 48 differences, which by comparison to the revised Cambridge Reference Sequence [28] and the PhyloTree website [29] revealed the subject to belong to mtDNA haplogroup J2a1a (coding region transitions including nucleotide positions 7789, 13722, 14133). The rather high number of differences is explained by the fact that the reference sequence belongs to the African haplogroup L3e2b1a (for example, differences at nucleotide positions 2483, 9377, 14905). Haplogroup J2a (formerly known as J1a) is only found at a frequency of approximately 0.3% in Ireland [30] but is ten times more common in Central Europe [31].

The distribution of this group has in the past been correlated with the spread of the Linearbandkeramik farming culture in the Neolithic [31], and maximum likelihood estimates of the age of J2a1 using complete mtDNA sequences give a point estimate of 7,700 years ago [32]; in good agreement with this thesis, sampled ancient mtDNA sequences from Neolithic sites in Central Europe predominantly belong to the N1a group [33].

### SNP imputation

The Irish population is of interest to biomedical researchers because of its isolated geography, ancestral impact on further populations and the high prevalence of a number of diseases, including cystic fibrosis, hemochromatosis and phenyketonuria [11]. Consequently, several disease genetic association studies have been carried out on Irish populations. As SNPs are often co-inherited in the form of haplotypes, such studies generally only involve genotyping subsets of known SNPs. Patterns of known co-inheritance, derived most commonly from the HapMap datasets, are then often used to infer the alleles at positions not directly typed using programs such as IMPUTE [34] or Beagle [35]. In the absence of any current or planned Irish-specific HapMap population, disease association studies have relied on the overall genetic proximity of the CEU dataset derived from European Americans living in Utah for use in such analyses. However, both this study (Figure 3) and previous work (Additional file 1) indicate that the Irish population is, at least to a certain extent, genetically distinct from the individuals that comprise the CEU dataset.

We were consequently interested in assessing the accuracy of genome-wide imputation of SNP genotypes using the previously unavailable resource of genome-wide SNP calls from our representative Irish individual. Using a combination of IMPUTE and the individual's genotype data derived from the SNP array we were able to estimate genotypes at 430,535 SNPs with an IMPUTE threshold greater than 0.9 (not themselves typed on the array). Within the imputed SNPs a subset of 429,617 genotypes were covered by at least one read in our analysis, and of those, 97.6% were found to match those called from the sequencing data alone.

**Figure 4:**
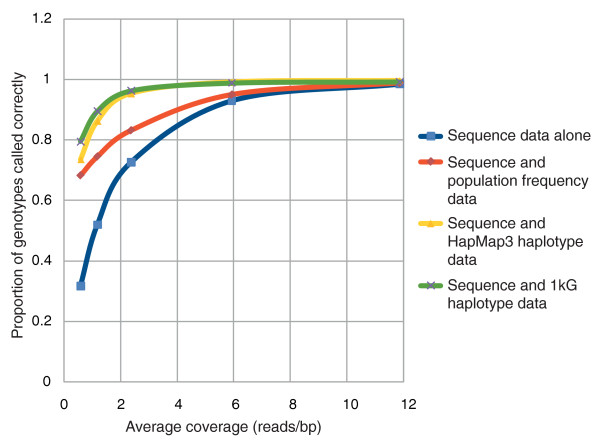
**Improved SNP calling using haplotype data**. SNP calling performance on chromosome 20 at various read depths with and without the inclusion of haplotype or genotype frequency data.

This successful application of imputation of unknown genotypes in our Irish individual prompted us to test whether haplotype information could also be used to improve SNP calling in whole genome data with low sequence coverage. Coverage in sequencing studies is not consistent, and regions of low coverage can be adjacent to those regions of relatively high read depth. As SNPs are often co-inherited, it is possible that high confidence SNP calls from well sequenced regions could be combined with previously known haplotype information to improve the calling of less well sequenced variants nearby. Consequently, we tested whether the use of previously known haplotype information could be used to improve SNP calling. At a given position where more than one genotype is possible given the sequencing data, we reasoned more weight should be given to those genotypes matching those we would expect given the surrounding SNPs and the previously known haplotype structure of the region. To test this, we assessed the improvements in SNP calling using a Bayesian approach to combining haplotype and sequence read information (see Materials and methods). Other studies have also used Bayesian methods to include external information to improve calls in low-coverage sequencing studies with perhaps the most widely used being SOAPsnp [36]. SOAPsnp uses allele frequencies obtained from dbSNP as prior probabilites for genotype calling. Our methods goes further, and by using known haplotype structures we can use information from SNPs called with relatively high confidence to improve the SNP calling of nearby positions. By comparing genotype calls to those observed on our SNP array we found substantial improvements can be observed at lower read depths when haplotype information is accounted for (Figure 4). At a depth of 2.4X, approximately 95% of genotypes matched those from the bead array when haplotype information was included, corresponding to the accuracy observed at a read depth of 8X when sequence data alone are used. Likewise, our method showed substantial improvements in genotype calling compared to only using previously known genotype frequency information as priors.

Given the comprehensive haplotype information likely to emerge from other re-sequencing projects and the 1000 Genomes project, our data suggest that sequencing at relatively low levels should provide relatively accurate genotyping data [37]. Decreased costs associated with lower coverage will allow greater numbers of genomes to be sequenced, which should be especially relevant to whole genome case-control studies searching for new disease markers.

### Causes of selection in the human lineage

There have been numerous recent studies, using a variety of techniques and datasets, examining the causes and effects of positive selection in the human genome [38-42]. Most of these have focused on gene function as a major contributing factor, but it is likely that other factors influence rates of selection in the recent human lineage. The availability of a number of completely sequenced human genomes now offers an opportunity to investigate factors contributing to positive selection in unprecedented detail.

Using this and other available completely sequenced human genomes, we first looked for regions of the human genome that have undergone recent selective sweeps by calculating Tajima's *D *in 10-kb sliding windows across the genome. Positive values of *D *indicate balancing selection while negative values indicate positive selection (see Materials and methods for more details). Due to the relatively small numbers of individuals from each geographical area (three Africans, three Asians and five of European descent - including reference) [16,26,43-48], we restricted the analysis to regions observed to be outliers in the general global human population.

A previous, lower resolution analysis using 1.2 million SNPs from 24 individuals and an average window size of 500-kb had previously identified 21 regions showing evidence of having undergone recent selective sweeps in the human lineage [41]. Our data also showed evidence of selection in close proximity to the majority of these regions (Table 3).

**Table 3:** Regions of high positive selection, in close proximity to genes, identified in the analysis of Williamson *et al*. [41]

**Williamson *et al***. [41]**regions of high positive selection**			Corresponding regions of low Tajima's D in this analysis		
	
Chr	Position (hg18)	Nearest gene	Position (hg18)	Nearest gene	Tajima's D
1	113519196	LRIG2 (50 kb)	113505001-113515000	-	-1.72
1	155990832	FCRL2 (0)	155990001-156000000	FCRL2 (0 kb)	**-2.08**
1	212654925	PTPN14 (0)	212595001-212605000	-	-1.09
2	140931201	LRP1B (0)	140930001-140940000	LRP1B (0 kb)	**-2.06**
2	201548002	MGC39518 (3 kb)	201455001-201465000	-	-1.73
3	29922879	RBMS3 (0)	29915001-29925000	RBMS3 (0 kb)	**-2.17**
3	43338322	SNRK (0)	43385001-43395000	-	-1.30
3	145075381	SLC9A9 (26 kb)	145090001-145100000	-	-1.71
4	71744283	IGJ (0)	71740001-71750000	IGJ (0 kb)	**-2.55**
4	169386385	FLJ20035 (0)	169395001-169405000	FLJ20035/DDX60 (0 kb)	**-2.10**
5	15527762	FBXL7 (26 kb)	15535001-15545000	FBXL7 (8.3 kb)	**-2.23**
6	128662923	PTPRK (0)	128655001-128665000	PTPRK (0 kb)	**-2.37**
8	57165523	RPS20 (16 kb)	57200001-57210000	PLAG1 (26 kb)	**-2.06**
10	45498260	ANUBL1 (10 kb)	45495001-45505000	FAM21C (0 kb)	**-2.27**
12	81525433	DKFZp762A217 (79 kb)	81520001-81530000	DKFZp762A217 (75 kb)	**-2.21**
13	37806830	UFM1 (15 kb)	37805001-37815000	-	-1.38
15	37639096	THBS1 (21 kb)	37640001-37650000	-	-1.95
15	89644996	SV2B (5 kb)	89640001-89650000	SV2B (0 kb)	**-2.08**
16	80605406	HSPC105 (3 kb)	80595001-80605000	-	-1.87
18	30388871	DTNA (0)	30380001-30390000	DTNA (0 kb)	**-2.21**
18	44274281	KIAA0427 (45 kb)	44365001-44375000	KIAA0427 (0 kb)	**-2.28**

Regions in this analysis with a Tajima's *D *value of less than -2 within 100 kb of the corresponding region from Williamson *et al*. [41] are highlighted in bold. (Selection of 21 random positions in the genome 1,000 times never produced as many within close proximity to a window whose Tajima's *D *was less than -2.)

### Gene pathways associated with selection in the human lineage

Examination of genes under strong positive selection using the GOrilla program [49] identified nucleic acid binding and chromosome organization as the Gene Ontology (GO) terms with the strongest enrichment among this gene set (uncorrected *P *= 2.31 × 10^-9 ^and 4.45 × 10^-8^, respectively).

Genes with the highest Tajima's *D *values, and predicted to be under balancing selection, were most enriched with the GO term associated with the sensory perception of chemical stimuli (uncorrected *P *= 2.39 × 10^-21^). These data confirm a previous association of olfactory receptors with balancing selection in humans using HapMap data [50]. However, our analysis also identified that a range of taste receptors were among the top genes ranked by *D *value, suggesting that balancing selection may be associated with a wider spectrum of human sensory receptors than previously appreciated.

The next most significantly enriched GO term, not attributable to the enrichment in taste and olfactory receptors, was keratinization (uncorrected *P *= 3.23 × 10^-5^) and genes affecting hair growth have previously been hypothesized to be under balancing selection in the recent human lineage [51].

### Gene duplication and positive selection in the human genome

Although most studies examine gene pathways when investigating what underlies positive selection in the human genome, it is likely other factors, including gene duplication, also play a role. It is now accepted that following gene duplication the newly arisen paralogs are subjected to an altered selective regime where one or both of the resulting paralogs is free to evolve [52]. Largely due to the lack of available data, there has been little investigation of the evolution of paralogs specifically within the human lineage. A recent paper has suggested that positive selection has been pervasive during vertebrate evolution and that the rates of positive selection after gene duplication in vertebrates may not in fact be different to those observed in single copy genes [53]. The emergence of a number of fully sequenced genomes, such as the one presented in this report, allowed us to investigate the rates of evolution of duplicated genes arising at various time points through the human ancestral timeline.

**Figure 5:**
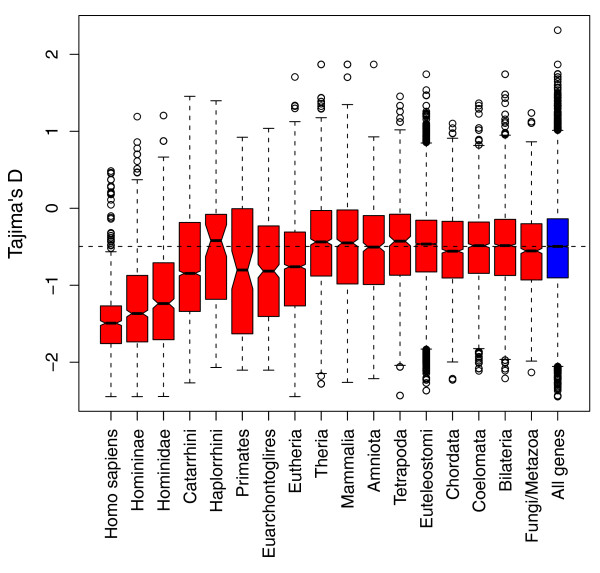
**Tajima's D values for paralogs arisen from gene duplications of different ages**. Mean Tajima's D values for genes involved in duplication events of differing ages. Horizontal dotted line indicates median Tajima's D value of all genes in human genome. As can be seen, genes involved in a recent duplication event in general show lower values of D than the genome-wide average, with genes involved in a duplication event specific to Humans, as a group, showing the lowest values of D. (Kruskal-Wallis *P *< 2.2 × 10^-16^).

As shown in Figure 5, there is clear evidence in our analysis for high levels of positive selection in recent paralogs, with paralogs arising from more recent duplication events displaying substantially lower values of Tajima's *D *than the background set of all genes. Indeed, elevated levels of positive selection over background rates are observed in paralogs that arose as long ago as the eutherian ancestors of humans (Figure 5). Consequently, while in agreement with the previous observation of no general elevation in the rates of evolution in paralogs arising from the most ancient, vertebrate duplication events, these data clearly illustrate that more recently duplicated genes are under high levels of positive selection.

As discussed, it has been proposed that, upon gene duplication, one of the gene copies retains the original function and is consequently under stronger purifying selection than the other. However, it has also been proposed that both genes may be under less sequence restraint, at least in lower eukaryotes such as yeast [52].

We consequently examined the rates of positive selection in both copies of genes in each paralog pair to see whether both, or just one, in general show elevated rates of positive selection in the human lineage. More closely examining paralog pairs that arose from a duplication event in *Homo sapiens *highlighted that even when only those genes in each paralog pair whose value of *D *was greater were examined, their *D *values were still significantly lower than the genome average (*t*-test *P *< 2.2 × 10^-16^), illustrating that even those genes in each paralog pair showing the least evidence of positive selection still show substantially higher levels of positive selection than the majority of genes. These results therefore support the hypothesis that both paralogs, rather than just one, undergo less selective restraint following gene duplication. Consequently, a significant driver for many of the genes undergoing positive selection in the human lineage (Table S3 in Additional file 2) appears to be this high rate of evolution following a duplication event. For example, 25% of those genes with a Tajima's *D *value of less than -2 have been involved in a duplication event in *Homo sapiens*, compared to only 1.63% of genes with *D *values greater than this threshold (chi-squared *P *< 2.2 × 10^-16^), illustrating that there is a substantial enrichment of genes having undergone a recent duplication event among the genes showing the strongest levels of positive selection. In conclusion, it appears that whether a gene has undergone a recent duplication event is likely to be at least as important a predictor of its likelihood of being under positive selection as its function.

## Conclusions

The first Irish human genome sequence provides insight into the population structure of this branch of the European lineage, which has a distinct ancestry from other published genomes. At 11-fold genome coverage, approximately 99.3% of the reference genome was covered and more than 3 million SNPs were detected, of which 13% were novel and may include specific markers of Irish ancestry. We provide a novel technique for SNP calling in human genome sequence using haplotype data and validate the imputation of Irish haplotypes using data from the current Human Genome Diversity Panel (HGDP-CEPH). Our analysis has implications for future re-sequencing studies and suggests that relatively low levels of genome coverage, such as that being used by the 1000 Genomes project, should provide relatively accurate genotyping data. Using novel variants identified within the study, which are in LD with already known disease-associated SNPs, we illustrate how these novel variants may point towards potential causative risk factors for important diseases. Comparisons with other sequenced human genomes allowed us to address positive selection in the human lineage and to examine the relative contributions of gene function and gene duplication events. Our findings point towards the possible primacy of recent duplication events over gene function as indicative of a gene's likelihood of being under positive selection. Overall, we demonstrate the utility of generating targeted whole-genome sequence data in helping to address general questions of human biology as well as providing data to answer more lineage-restricted questions.

## Materials and methods

### Individual sequenced

It has been recently shown that population genetic analyses using dense genomic SNP coverage can be used to infer an individual's ancestral country of origin with reasonable accuracy [15]. The sample sequenced here was chosen from among a cohort of 211 healthy Irish control subjects included in recent genome-wide association studies [13,14] with all participants being of self-reported Irish Caucasian ethnicity for at least three generations. Using Illumina Infinium II 550 K SNP chips, the Irish samples were assayed for 561,466 SNPs selected from the HapMap project. Quality control and genotyping procedures have been detailed previously [15]. We have previously published 300 K density STRUCTURE [54,55] and principle components analyses of the Irish cohort both in comparison to similar cohorts from the UK, Netherlands, Denmark, Sweden and Finland [15], and in separate analyses in comparison to additional cohorts from the UK, Netherlands, Sweden, Belgium, France, Poland and Germany [14]. The data demonstrate a broad east-west cline of genetic structure across Northern Europe, with a lesser north-south component [15]. Individuals from the same populations cluster together in these joint analyses. Using these data, we here selected a 'typical' Irish sample, which clustered among the Irish individuals and was independent of the British samples, for further characterization.

### Genomic library preparation and sequencing

All genomic DNA libraries were generated according to the protocol Genomic DNA Sample Prep Guide - Oligo Only Kit (1003492 A) with the exception of the chosen fragmentation method. Genomic DNA was fragmented in a Biorupter™ (Diagenode, Liége, Belgium). Paired-end adapters and amplification primers were purchased from Illumina (Illumina, San Diego, CA, USA catalogue number PE-102-1003). New England Biolabs (New England Biolabs, Ipswich, MA, USA) was the preferred supplier for all enzymes and buffers and Invitrogen (Invitrogen, Carlsbad, CA, USA) for the dATP. Briefly, the workflow for library generation was as follows: fragmentation of genomic DNA; end repair to create blunt ended fragments; addition of 3'-A overhang for efficient adapter ligation; ligation of the paired-end adapters; size selection of adapter ligated material on a 2.5% high resolution agarose (Bioline HighRes Grade Agarose - Bioline, London, UK), catalogue number BIO-41029); a limited 12 cycle amplification of size-selected libraries; and library quality control and quantification. For each library 5 μg of DNA was diluted to 300 μl and fragmented via sonication - 30 cycles on Biorupter High setting with a cycle of 30 s ON and 30 s OFF. All other manipulations were as detailed in the Illumina protocol.

Quantification prior to clustering was carried out with a Qubit™ Fluorometer (Invitrogen Q32857) and Quant-iT™ dsDNA HS Assay Kit (Invitrogen Q32851). Libraries were sequenced on Illumina GAII and latterly GAIIx Analyzer following the manufacturer's standard clustering and sequencing protocols - for extended runs multiple sequencing kits were pooled.

### Read mapping

NCBI build 36.1 of the human genome was downloaded from the UCSC genome website and the bwa alignment software [56] was used to align both the single- and paired-end reads to this reference sequence. Two mismatches to the reference genome were allowed for each read. Unmapped reads from one single-end library were trimmed and remapped due to relative poor quality at the end of some reads, but none were trimmed shorter than 30 bp.

### SNP and indel identification

SNPs were called using samtools [57] and glfProgs [58] programs. The criteria used for autosomal SNP calling were: 1, a prior heterozygosity (theta) of 0.001; 2, positions of read depths lower than 4 or higher than 100 were excluded; 3, a Phred-like consensus quality cutoff of no higher than 100.

Only uniquely mapped reads were used when calling SNPs. SNPs in the pseudoautosomal regions of the X and Y chromosomes were not called in this study and consequently only homozygous SNPs were called on these chromosomes. The criteria used for sex chromosome SNP calling were: 1, positions of read depths lower than 2 or higher than 100 were excluded; 2, the likelihoods of each of the four possible genotypes at each position were calculated and where any genotype likelihood exceeded 0.5 that did not match the reference a SNP was called.

The positive predictive value in our study, assessed using the 550 k array data as in other studies [48], was 99%. As a result of maintaining a low false positive rate, the heterozygote undercall rate observed in this analysis was slightly higher than in other studies of similar depth - 26% as opposed to 24% and 22% in the Watson and Venter genomes, respectively.

SNP consequences were determined using the Ensembl Perl APIs and novel SNPs identified through comparisons with dbSNP130 obtained from the NCBI ftp site. Further human genome SNP sets were also downloaded from their respective sources [7,16,26,43-48]. The CEU dataset for the SNP imputation and population structure analysis were downloaded from the Impute and HapMap websites, respectively. Previously identified disease variants were downloaded from OMIM (15 April 2009) and HGMD (HGMD Professional version 2009.4 (12 November 2009)). Pairs of HapMap SNPs in high LD flanking novel markers and known disease variants were identified using the Ensembl Perl APIs.

Indels were called using samtools [57]. Short indels had to be separated by at least 20 bp (if within 20 bp, the indel with the higher quality was kept) and for the autosomes had to have a mapping quality of greater than 20 and be covered by a read depth of greater than 4 and less than 100. For the sex chromosomes the lower threshold was set at 2. As with SNP calling, only uniquely mapped reads were used. Twenty-six randomly selected coding indels were confirmed via resequencing of which 24 displayed traces supporting the indel call. Of the remaining two, one showed a double trace throughout suggestive of unspecific sequencing, while the second showed no evidence of the indel (Table S4 in Additional file 2).

SNPs and indels were analyzed with SIFT tools at the J Craig Venter Institute website [59]. Indel positions were remapped to build 37 of the reference genome using the liftover utility at UCSC as a number of coding indels identified in build 36 were found not to affect corresponding genes when the latest gene builds were used. The identification of the enrichment of allele changes deemed by SIFT to be deleterious among novel SNPs in putative LD with disease markers was determined using both high and low confidence SIFT predictions of deleterious variants. However, when only the proportion of non-synonymous SNPs called deleterious with high confidence across the whole genome (744 out of 7,993; 9.3%) was compared to the number observed in the subset of SNPs in putative LD with disease markers (6 out of 25; 26.1%), a significant difference was still observed (*P *= 0.025, Fisher's exact test).

### Y chromosome analysis

All called Y chromosome nucleotide differences from the Human Reference sequence were catalogued. Although originating from multiple individuals, the majority of the Y chromosome reference sequence represents a consensus European R1b individual, either because all individuals in the pool belonged to this group, or because they outnumbered the others in the original sequencing. While most of the differences from the reference were novel, they included S145, which reaches frequencies of about 80% in Ireland. There are at present five known non-private subgroups of R1b-S145 (M222, S168, S169, S175 and S176, all seen in Ireland); none of these SNPs were identified in the Irish individual and he potentially belongs to an as yet undescribed sublineage within S145.

### Imputation

IMPUTE [34] version 1 was used in all imputation analyses and phased haplotype information for the 1000 Genomes project and HapMap3 release 2 were obtained from the IMPUTE website [60]. The accuracy of imputation in the Irish population was assessed using the genotypes from the Illumina bead array and the HapMap 3 haplotypes [20]. Only genotypes at SNPs not on the bead array with an IMPUTE score above 0.9 were compared to the most probable genotype from the sequencing data obtained with glfProgs. Where more than one genotype was equally likely, one was chosen at random.

In an attempt to improve SNP calling, haplotype information was combined with sequencing data via a Bayesian approach. At any given position in the genome, 1 of 16 genotypes must be present (AA, AT, AC, AG, TT, TC and so on) and glfProgs provides the likelihood ratio for each of these possible genotypes at each position given the observed sequence data. The likelihood ratio is defined as the likelihood ratio of the most likely genotype to the genotype in question and consequently the likelihood ratio of the most likely genotype will be 1. As there are only 16 possible genotypes, it is possible to obtain the likelihood for each genotype at each position by dividing the genotype's likelihood ratio by the sum of all 16 likelihood ratios at that position, giving our conditionals.

To calculate our genotype priors at any given position in the genome, we took the probabilities of the genotypes at surrounding positions in the genome (obtained from the sequencing data alone using glfProgs as described above) and used these as input to the IMPUTE program to predict the probabilities of each genotype at the position of interest, giving our priors. Posteriors were then calculated using the standard Bayes formula.

To assess the effectiveness of imputation-based priors at various coverage depths, mapped reads were randomly removed and the above process repeated (the resulting genotype calls for chromosome 20 are provided in Additional file 5).

### Selection

Tajima's *D *values for each 10-kb window of the human genome were calculated using the variscan software [61], with a 5-kb overlap between adjacent windows. Tajima's *D *compares two estimates of the population genetics parameter θ; namely, the average number of differences seen between each pair of sequences (θ_w_) and the observed number of segregating sites (θ_S_) [62]. When a population evolves neutrally these two values are expected to be approximately equal. If, however, a region is under positive selection, mutations at this location would be expected to segregate at lower frequencies, leading to a lower observed average number of differences between each pair of sequences (θ_w_). On the other hand, under balancing selection this average number of differences will be expected to be larger. By comparing θ_w _to θ_S _it is possible to determine regions of selection, the principle underlying Tajima's *D*. Where positive selection is occurring θ_w _will be small and Tajima's *D *will be negative, while balancing selection will lead to larger values of θ_w _and positive values of *D*. In this analysis ten re-sequenced genomes were used; the Irish sample described here, three further Caucasians (NA07022, Watson and Venter), one Chinese, two Koreans, and three Africans (only the Bantu genome from [16] was included as, unlike the Khoisan genome, SNP calls without the exome sequencing data were available, more closely corresponding to the datasets of the other genomes used) [16,26,43-48]. Consequently, along with the haploid reference genome, a total of 21 chromosomes were used in this analysis. As in previous studies [63] we used a cutoff of -2 to indicate putative regions of positive selection and +2 to indicate putative regions of balancing selection. In total 9,152 (1.6%) of the 573,533 overlapping windows in the genome had a *D *value of less than -2 in our analysis, corresponding to 4,819 distinct regions (having concatenated overlapping windows).

The coordinates of Williamson *et al*.'s [41] regions of high positive selection were converted to build 36 positions through the use of the liftover utility at UCSC. The analysis of Williamson *et al*. had shown that regions close to centromeres often display high levels of recent selection and the regions identified in our study as showing the strongest evidence of having undergone recent selective sweeps were also overwhelmingly located at chromosomal centromeres (data not shown). Consequently, despite our relatively small number of individuals, our high number of SNPs gave us the power to detect previously identified regions of selection even when a small window size was used, allowing us to pick up regions with a finer resolution than has been possible in previous analyses.

Average Tajima's *D *values were calculated for each Ensembl 54 protein coding gene by averaging the corresponding values for all windows that it overlapped. Ranked GO enrichment analysis was carried out using the GOrilla application [49]. The list of paralogs used in this analysis, and their associated age, were obtained from Vilella *et al*. [64]. Paralogs in close proximity (< 250 kb) were ignored.

### Population structure

The AWclust R package [25] was used for the non-parametric population structure analysis. Only unrelated members of the CEU HapMap dataset were retained in the analysis, all trio offspring being excluded. We used 405,737 autosomal SNPs from the Illumina 550 k set for which genotypes were present for all individuals in this analysis. Information from the sequence of NA07022 was not included due to his presence in the HapMap dataset.

### Data accessibility

The sequence data from this study have been linked to the expression study cited in the manuscript under the dbGap accession [dbGap:phs000127.v2.p1] and deposited in the NCBI Short Read Archive [65] under study accession preferred accession number [SRA:SRP003229]. The SNPs and indels have been submitted to NCBI dbSNP and will be available in dbSNP version B133. The data have also been submitted to Galaxy [66].

## Abbreviations

bp: base pair; GO: Gene Ontology; HGMD: Human Gene Mutation Database; LD: linkage disequilibrium; mtDNA: mitochondrial DNA; OMIM: Online Mendelian Inheritance in Man; SNP: single nucleotide polymorphism.

## Authors' contributions

AL performed the experiments; PT, JW, NF and JP performed the data analysis; JP, PT and BL wrote the manuscript; all of the authors contributed to the research design, discussed the results and commented on the manuscript.

## Supplementary Material

Additional file 1 **Figure S1**. Principal components analysis plot adapted from [15] illustrating the position of our Irish Individual with respect to other individuals of western European origin. Click here for file

Additional file 2 **Supplementary tables**. Table S1: novel variants in LD with heterozygous polymorphisms previously associated with disease. Table S2: indels in coding sequence regions. Table S3: Tajima's *D *values. Table S4: re-sequencing results of 26 coding indels. Click here for file

Additional file 3 **Figure S2. **Confirmation of rs3197999 in the Irish individual via standard PCR resequencing. Click here for file

Additional file 4 **Figure S3**. Confirmation of the novel nonsense variant in *MST1 *via standard PCR followed by sequencing. Click here for file

Additional file 5 **Table S5. **The resulting genotype calls for chromosome 20. Click here for file
