# Supplementary figures and images for: Sequencing and analysis of an Irish human genome
Source: Genome Biol. 2010 Sep 7;11(9):R91. doi: 10.1186/gb-2010-11-9-r91 (PMC2965383; doi:10.1186/gb-2010-11-9-r91)

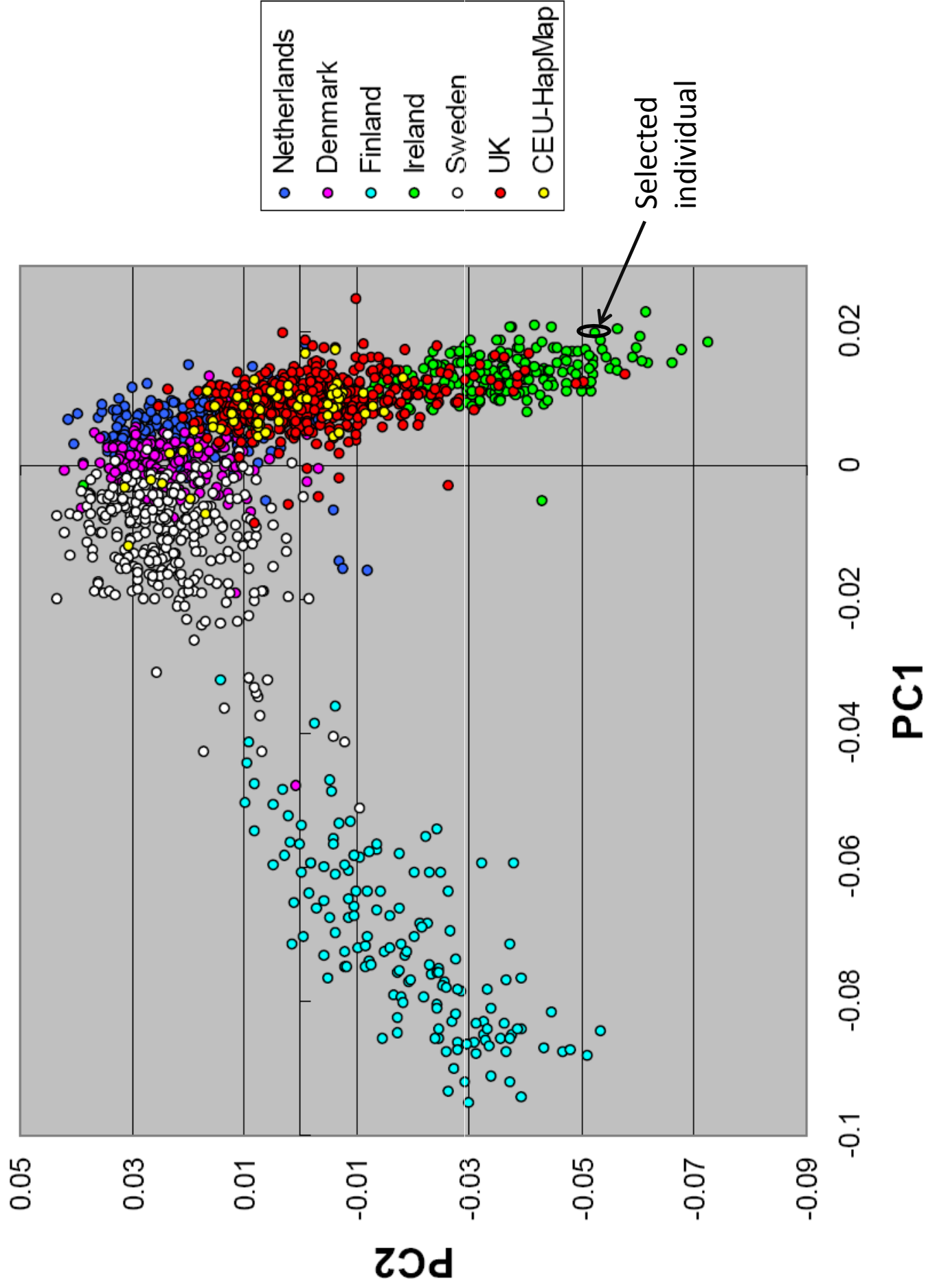

Figure adapted from [13] with the kind permission of Dr B McEvoy

Supplement: Additional file 1 — Figure S1. Principal components analysis plot adapted from [15] illustrating the position of our Irish Individual with respect to other individuals of western European origin. [file gb-2010-11-9-r91-S1.PDF]

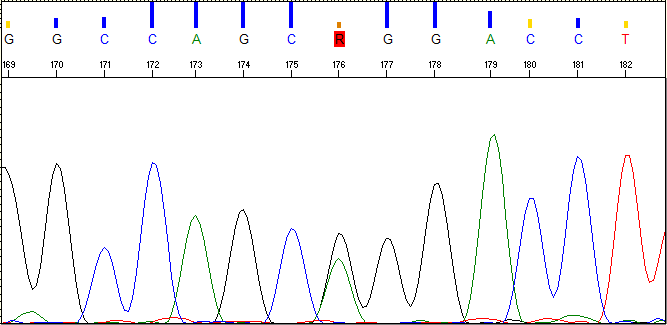

Supplement: Additional file 3 — Figure S2. Confirmation of rs3197999 in the Irish individual via standard PCR resequencing. [file gb-2010-11-9-r91-S3.PNG]

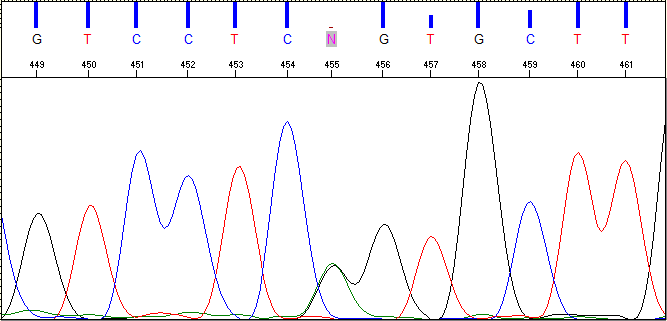

Supplement: Additional file 4 — Figure S3. Confirmation of the novel nonsense variant in MST1 via standard PCR followed by sequencing. [file gb-2010-11-9-r91-S4.PNG]
